# What will we see in diabetes in the next 10 years?

**DOI:** 10.1111/1753-0407.13594

**Published:** 2024-06-18

**Authors:** Zachary T. Bloomgarden

**Affiliations:** ^1^ Department of Medicine, Division of Endocrinology Diabetes and Bone Disease, Icahn School of Medicine at Mount Sinai New York NY USA

The theme of growing numbers of persons with diabetes in relation to the aging of the population and to worsening obesity are factors brought up by Ellliott Joslin more than six decades ago in an article entitled “Diabetes in the Future”^1^—but it remains daunting to face the reality of a progressive threefold increase from approximately 150 million in 2000 to more than 500 million cases today, with projections of a total of 700–800 million persons with diabetes by 2045.^2^ The greatest increases will be in South‐East Asia, from 90 to over 150 million, and in the Middle East and North Africa, from approximately 70 to 140 million, albeit with the Western Pacific countries increasing from 200 million to 260 million persons to remain the largest region of persons with diabetes.^3^

Diabetes prevalence increases with increasing age and increasing degrees of obesity, with both of these factors projected to play important roles in the growth of diabetes over the coming decades. The prevalence of diabetes increases from <5% before the age of 35 to 5%–10% from age 35–50 and to nearly 15% in middle‐income countries, remaining around 10% from age 50–80 in low‐income countries but increasing to >20% in middle‐income countries and to >25% in high‐income countries at ages 65 and over.^2^ This becomes particularly important when we consider world population trends. The United Nations' projection of World Population Prospects showed that in 1950, there were fewer than 200 million persons aged 65 and over; this number began to increase gradually and by 2000, reached approximately 500 million, with projections that in the year 2100, there will be approximately 2.5 billion persons in this age group, equaling the number of persons aged less than 20 and 45–64, with the number of persons aged 20–44 being relatively static at an additional 3.5 billion.^4^ The same dataset shows that in China, the largest part of the Western Pacific region, the number of persons aged 20–44 is likely to have peaked around 2010, with projections of a decline in this age group from nearly 600 billion to approximately 300 billion in the year 2100, equaling the number of persons aged 65 and over and exceeding that in the 45–65‐year‐old and <20‐year‐old age groups.^4^ Taken together, the higher prevalence of diabetes with increasing age along with the increasing numbers of persons at greater ages implies that older persons contribute disproportionately to the population with diabetes, comprising a proportion increasing from 30% to 40% of persons with diabetes in the United States from 1980 to 2010.^5^

In addition to the aging of the population, the growth in numbers of persons with increasing degrees of obesity is an evident and worrisome characteristic; virtually every high‐income country is seeing increases in prevalence of obesity, with annual data from the United States, England, Spain, Austria, Australia, France, Korea, Canada, and Italy each showing nearly linear trends from 30%–50% to 40%–70% of the population being overweight or obese.^6^ Studies in the United States show state‐by‐state obesity prevalence increasing every decade from 1990 to projected levels in 2030, with most states having 20%–25% obesity prevalence in 1990, now increasing to 40%–50% and with the prevalence of severe obesity increasing from <15% to 20%–30%.^7^ A similar analysis of obesity and abdominal obesity prevalence in China from 2015 to 2030 again shows linear increases.^8^ In turn, obesity has played a major role in the increasing numbers of persons with diabetes. This is most readily perceived in comparisons of prevalences of diabetes with those of obesity in different countries, with data from 183 countries in 2014 showing that those countries with obesity prevalence of 10% or lower have diabetes prevalence around 10%, while as obesity prevalence in other countries increased from 30% to 60%, diabetes prevalence increased from 20% to 30%; projections from this study suggest that in 2030, these countries can expect increases both in the degree of obesity and the prevalence of diabetes.^9^

The inevitable result of the growing prevalence of diabetes will be the increasing levels of complications. Globally, diabetic retinopathy is projected to affect 160 million persons by 2045, with greatest projected prevalences in North America, South‐East Asia, Middle East and North Africa, and the Western Pacific region.^10^ Cardiovascular disease risk factors in the United States over the coming three to four decades will include not only diabetes increasing by 39.3% to 55 million persons but also increases in hypertension and dyslipidemia, with projections that, across the population, ischemic heart disease will increase by 30.7%, heart failure by 33.4%, myocardial infarction by 16.9%, and stroke by 33.8%.^11^ Diabetes is strongly associated with cancer,^12^ chronic kidney disease,^13^ dementia,^14^ and pulmonary disease,^15^ with obesity as an important mediator of all these complications.^16^

What are some of the approaches that can be taken to address this growing problem? Despite the strong association of diabetes with atherosclerosis, recent population studies in the United States show low use of cardioprotective therapies, with only one quarter of persons with diabetes receiving high‐dose statins, less than half receiving angiotensin‐directed agents, and only 3% and 4% receiving Sodium‐glucose countertransporter2 inhibitor (SGLT2i) and Glucagon‐like Peptide‐1 receptor agonist (GLP‐1RA) treatment, respectively.^17^ Certainly, ongoing attention to effective glycemic control is crucial. The recent 24‐year follow‐up from the United Kingdom Prospective Diabetes Study (UKPDS) reports that there were “near‐lifelong legacy effects of early intensive glycaemic control with sulfonylurea or insulin and with metformin … near‐normal glycaemia immediately after type 2 diabetes is diagnosed appears to be essential”; >30‐year analysis of patterns of myocardial infarction show overall rates of 18.4% versus 21.7%, comparing sulfonylurea/insulin treatment with what was at the time of the initial UKPDS considered “conventional” treatment and 17.3% versus 23.4%, comparing metformin with its control group.^18^ A projection of data from Australia suggests that the combination of SGLT2i treatment with lifestyle interventions could lead to a 20% reduction in diabetes‐associated end‐stage renal disease development.^19^ Similar analysis based on US data estimates lifetime atherosclerotic cardiovascular disease, renal, and mortality benefits of combined use of SGLT2i, GLP‐1RA, and nonsteroidal mineralocorticoid receptor antagonists on the order of 35%–55%.^20^ Not only better delivery of medication but also greater use of technology has the promise to improve outcome, aiding patient communication with telemedicine and beyond and addressing diet, exercise, and tracking of blood pressure, body weight, and blood glucose, with the promise of large language artificial intelligence models supplementing the interaction of patients with healthcare providers.

Our challenge is to effectively deliver these treatments to the growing numbers of patients with diabetes.
